# Acute spatial spread of NO‐mediated potentiation during hindpaw ischaemia in mice

**DOI:** 10.1113/JP277615

**Published:** 2019-05-28

**Authors:** Takeshi Onishi, Tatsunori Watanabe, Mika Sasaki, Yoshinori Kamiya, Masao Horie, Hiroaki Tsukano, Ryuichi Hishida, Tatsuro Kohno, Hirohide Takebayashi, Hiroshi Baba, Katsuei Shibuki

**Affiliations:** ^1^ Department of Neurophysiology Brain Research Institute Niigata University Niigata 951‐8585 Japan; ^2^ Department of Anesthesiology Faculty of Medicine, Niigata University Niigata 951‐8510 Japan; ^3^ Department of Morphological Sciences Faculty of Medicine, Kagoshima University Kagoshima 890‐8544 Japan; ^4^ Department of Anesthesiology Faculty of Medicine, Tohoku Medical and Pharmaceutical University Sendai 983‐8536 Japan; ^5^ Department of Neurobiology and Anatomy Faculty of Medicine, Niigata University Niigata 951‐8510 Japan

**Keywords:** Neuropathic pain, Nitric oxide, Spinal cord

## Abstract

**Key points:**

- Neuropathic pain spreads spatially beyond the injured sites, and the mechanism underlying the spread has been attributed to inflammation occurring in the spinal cord.
- However, the spatial spread of spinal/cortical potentiation induced by conduction block of the peripheral nerves can be observed prior to inflammation.
- In the present study, we found that spreading potentiation and hypersensitivity acutely induced by unilateral hindpaw ischaemia are nitric oxide (NO)‐dependent and that NO is produced by ischaemia and quickly diffuses within the spinal cord.
- We also found that NO production induced by ischaemia is not observed in the presence of an antagonist for group II metabotropic glutamate receptors (mGluRs) and that neuronal NO synthase‐positive dorsal horn neurons express group II mGluRs.
- These results suggest strongly that NO‐mediated spreading potentiation in the spinal cord is one of the trigger mechanisms for neuropathic pain.

**Abstract:**

Cortical/spinal responses to hindpaw stimulation are bilaterally potentiated by unilateral hindpaw ischaemia in mice. We tested the hypothesis that hindpaw ischaemia produces nitric oxide (NO), which diffuses in the spinal cord to induce spatially spreading potentiation. Using flavoprotein fluorescence imaging, we confirmed that the spreading potentiation in hindpaw responses was induced during ischaemia in the non‐stimulated hindpaw. This spreading potentiation was blocked by spinal application of l‐NAME, an inhibitor of NO synthase (NOS). Furthermore, no spreading potentiation was observed in neural NOS (nNOS) knockout mice. Spinal application of an NO donor was enough to induce cortical potentiation and mechanical hypersensitivity. The spatial distribution of NO during unilateral hindpaw ischaemia was visualized using 4‐amino‐5‐methylamino‐2′,7′‐difluorofluorescein (DAF‐FM). An increase in fluorescence derived from the complex of DAF‐FM with NO was observed on the ischaemic side of the spinal cord. A similar but smaller increase was also observed on the contralateral side. Somatosensory potentiation after hindpaw ischaemia is known to be inhibited by spinal application of LY354740, an agonist of group II metabotropic glutamate receptors (mGluRs). We confirmed that the spinal DAF‐FM fluorescence increases during hindpaw ischaemia were not observed in the presence of LY354740. We also confirmed that approximately half of the nNOS‐positive neurons in the superficial laminae of the dorsal horn expressed *mGluR2* mRNA. These results suggest that disinhibition of mGluR2 produces NO which in turn induces a spreading potentiation in a wide area of the spinal cord. Such spreading, along with the consequent non‐specific potentiation in the spinal cord, may trigger neuropathic pain.

## Key points

- Neuropathic pain spreads spatially beyond the injured sites, and the mechanism underlying the spread has been attributed to inflammation occurring in the spinal cord.
- However, the spatial spread of spinal/cortical potentiation induced by conduction block of the peripheral nerves can be observed prior to inflammation.
- In the present study, we found that spreading potentiation and hypersensitivity acutely induced by unilateral hindpaw ischaemia are nitric oxide (NO)‐dependent and that NO is produced by ischaemia and quickly diffuses within the spinal cord.
- We also found that NO production induced by ischaemia is not observed in the presence of an antagonist for group II metabotropic glutamate receptors (mGluRs) and that neuronal NO synthase‐positive dorsal horn neurons express group II mGluRs.
- These results suggest strongly that NO‐mediated spreading potentiation in the spinal cord is one of the trigger mechanisms for neuropathic pain.

## Introduction

Peripheral nerve injuries produce neuropathic pain in animal models (Bennett & Xie, 1988; Kim & Chung, 1992; Decosterd & Woolf, 2000). Neuropathic pain develops chronically over 2–3 weeks after the peripheral nerve injury, and is modified by inflammatory responses occurring in the spinal cord and by the resulting epigenetic regulation (Campbell & Meyer, 2006; Penas & Navarro, 2018). At the same time, somatosensory cortical plasticity is acutely induced after nerve injuries (Calford & Tweedale, 1988) and nerve conduction block (Björkman *et al*. 2009). Acute cortical potentiation occurs within several hours after partial nerve cutting and before the inflammatory responses occur; therefore, it may be the initial pathological step for the development of neuropathic pain (Komagata *et al*. 2011). An abnormal sensation of irritation frequently occurs after the ischaemic conduction block of peripheral nerves (Mogyoros *et al*. 2000), and hindpaw ischaemia and the resulting conduction block of peripheral nerves induce cortical/spinal potentiation within 30 min (Watanabe *et al*. 2015). Therefore, it is natural to assume that similar neural mechanisms are responsible for both types of acute potentiation after partial nerve cutting and ischaemic conduction block. Because hindpaw ischaemia for 3 h is enough to induce neuropathic pain lasting for 4 weeks (Coderre *et al*. 2004), the neural mechanisms that trigger neuropathic pain might be understood by investigating the acute neural changes that occur during hindpaw ischaemia.

Cortical potentiation after partial nerve cutting is observed in uninjured nerves (Komagata *et al*. 2011). Similarly, spatial spreading is observed in the potentiation that follows hindpaw ischaemia: potentiation is induced on the contralateral side of ischaemic conduction block, as well as on the ipsilateral side (Watanabe *et al*. 2015). Spatial spreading is also a characteristic feature of neuropathic pain: it is observed in sites distant from the site of nerve injury (Milligan *et al*. 2003; Racz *et al*. 2008; Shenker *et al*. 2008; Carlton *et al*. 2009). Spatial invasion of the inflammatory responses within the spinal cord may be responsible for the spread of information between a particular spinal neuron that fails to receive afferents and the surrounding spinal neurons that receive normal afferents. It is also possible that the spreading information may be distributed via neural networks (Fitzgerald, 1982; Koltzenburg *et al*. 1999) or diffusion of chemical mediators. The mouse may be an ideal experimental animal model for testing the third possibility, because the mouse spinal cord is sufficiently small that influences of unilaterally produced diffusible mediators can be detected on the other side of the spinal cord (Watanabe *et al*. 2015).

Nitric oxide (NO) is a diffusible mediator involved in synaptic plasticity (Garthwaite & Boulton, 1995; Garthwaite, 2016) and neuropathic pain (Schmidtko *et al*. 2009). Neuronal NO synthase (nNOS) is distributed in the superficial laminae of the spinal dorsal horn (Zhang *et al*. 1993; Reuss & Reuss, 2001). Therefore, we tested the hypothesis that the spreading potentiation induced by unilateral hindpaw ischaemia is mediated by NO. It is also known that group II metabotropic glutamate receptors (mGluRs) are involved in the potentiation after partial nerve cutting (Komagata *et al*. 2011) and ischaemic conduction block (Watanabe *et al*. 2015), as in neuropathic pain (Simmons *et al*. 2002; Jones *et al*. 2005; Osikowicz *et al*. 2013). A simple explanation for the relationship between NO signalling and group II mGluRs is that the role of group II mGluRs on cortical/spinal potentiation is mediated by NO production. Therefore, we tested the effects of an agonist of group II mGluRs on spinal NO production. We also investigated whether group II mGluRs and nNOS are co‐localized in the same spinal neurons.

## Methods

### Ethical approval

The experiments in the present study were approved by the ethics committee for animal experiments in Niigata University (approved number: 233‐4 and 372‐7) and were carried out in accordance with the approved guidelines and the policy and regulations on animal experimentation of *The Journal of Physiology*.

### Animals

Male C57BL/6 mice of 7–10 weeks of age, purchased from Charles River Japan (Yokohama, Japan), were used in the present study. Male and age‐matched mice with a targeted disruption of the *nNOS* gene (Huang *et al*. 1993) obtained from The Jackson Laboratory (Bar Harbor, ME, USA) and bred in our laboratory were also used for the experiments.

### Surgery

Mice were operated on as described previously (Watanabe *et al*. 2015). They were anaesthetized with urethane (1.65 g/kg, i.p.), and a tracheotomy was performed to facilitate respiration. Body temperature was monitored using a rectal probe and maintained at 38°C using a silicon rubber heater. These surgical operations were usually completed within 60 min. Recordings were started 30 min after the surgical operations. Additional doses of urethane (0.1–0.2 g/kg, s.c.) were administered when necessary. To investigate the cortical responses to hindpaw stimulation, the disinfected head skin of the mice was incised, and the skull over the right somatosensory cortex was exposed. The surface of the skull was cleaned with sterile saline, and a small piece of metal was attached to the skull with a dental acrylic resin (Super Bond; Sun Medical, Shiga, Japan) to fix the head under a binocular epifluorescence microscope (M165 FC; Leica Microsystems, Wetzlar, Germany). The surface of the skull was covered with a mixture of petroleum jelly and liquid paraffin to keep the skull transparent. When spinal responses to hindpaw stimulation were investigated, the vertebral arch was removed at the T13 and L1 level, and the dorsal surface of the spinal cord with the intact dura mater was exposed. The surface was cleaned with saline, and covered with 2% agarose to prevent spinal movement. The surface of the agarose gel was covered with a mixture of petroleum jelly and liquid paraffin to prevent drying. The spinal cord was fixed under the binocular epifluorescence microscope using a clamp (STS‐A; Narishige, Tokyo, Japan). Spontaneous respiration was maintained during the imaging experiment, because movement of the spinal cord caused by respiration is typically minimal.

### Flavoprotein fluorescence imaging

Flavoprotein fluorescence imaging was performed to investigate the cortical and spinal responses to hindpaw stimulation, as previously described (Watanabe *et al*. 2015). Brush vibration with an amplitude of 0.2 mm at 50 Hz was applied for 600 ms to the sole of the hindpaw using a solenoid mechanical stimulator (DPS‐290; Dia Medical, Tokyo, Japan). Endogenous green fluorescence (wavelength: 500–550 nm) was recorded in blue light (wavelength: 450–490 nm). Images (128 × 168 pixels) of the primary somatosensory cortex or the spinal cord were recorded at 9 frames/s using a cooled charge coupled device camera (ORCA‐R2; Hamamatsu Photonics, Hamamatsu, Japan). The camera was attached to the binocular epifluorescence microscope with a 75‐W xenon light source and a 1× objective lens. Serial images were taken in recording sessions repeated at 50‐s intervals. Fluorescence changes elicited by the stimulation were averaged over 24 trials. Because approximately 20 min was needed to obtain data from the 24 trials, the recording time of the averaged data was defined as the middle point of the recording period. Spatial averaging in 5 × 5 pixels and temporal averaging in three consecutive frames were used to smoothen and improve the image quality. The images were normalized, pixel by pixel, with respect to a reference image (*F*_0_), which was obtained by averaging five images taken immediately before stimulation. In the figures, the selected parts of the normalized images are shown in a pseudocolour scale representing the fractional fluorescence changes (Δ*F*/*F*_0_). The response amplitude of the cortical responses was evaluated with respect to Δ*F*/*F*_0_ in a square window of 60 × 60 pixels or 1.55 × 1.55 mm at 0.6–1.0 s after the onset of the stimulus. The response amplitude in spinal responses was evaluated with respect to Δ*F*/*F*_0_ in a square window of 100 × 25 pixels or 3.84 × 0.96 mm at 0.6‐1.0 s after the stimulus onset. The location of the window was adjusted to maximize the response amplitude with respect to Δ*F*/*F*_0_. After the recordings were completed, the mice were killed with an overdose of pentobarbital (300 mg/kg, i.p.).

### Hindpaw ischaemia

A small rubber cuff was set around the right thigh and covered with a hard‐plastic tube, as previously described (Watanabe *et al*. 2015). Air pressure at 250 mmHg was applied for 2 h to the tubing connected to the cuff using a mercury manometer. The pressure was directed to the thigh, because inflation of the rubber cuff was limited by the hard‐plastic tube. When the effects of a transient ischaemia were tested, the rubber cuff was set around the left thigh, and an air pressure of 250 mmHg was applied for 30 min. We confirmed that this pressure was sufficient to eliminate cortical responses induced by hindpaw stimulation during the ischaemia.

### Visualization of NO production in the spinal cord during hindpaw ischaemia

To visualize NO production in the spinal cord, 4‐amino‐5‐methylamino‐2′,7′‐difluorofluorescein (DAF‐FM; Goryo Chemical, Sapporo, Japan) was used (Kojima *et al*. 1999). DAF‐FM is converted to a fluorescent substance when combined with NO. The surface of the spinal cord was exposed at the T13–L1 level, and fixed under the same binocular epifluorescence microscope used for flavoprotein fluorescence imaging. To avoid the influence of the surgical operation, the surface of the spinal cord was coated with a 2% agarose gel containing 20 μM DAF‐FM 30 min after the operation. An image (256 × 336 pixels, exposure time: 500 ms) of the spinal cord was recorded in green fluorescence (wavelength: 500–550 nm) excited with blue light (wavelength: 450–490 nm) before the ischaemic treatment. Then, the ischaemic treatment was applied to the right hindpaw, and images of the spinal cord were acquired at 30‐min intervals during the hindpaw ischaemia. The obtained data were quantified by selecting a region of interest (ROI) of 100 × 20 pixels or 1.92 × 0.38 mm on the right ischaemic side and the left non‐ischaemic side, respectively. The position of the right ROI was determined so that the amount of increase in fluorescence intensity at 120 min after the onset of ischaemia was maximized in the ROI, and the left ROI on the non‐ischaemic side was positioned symmetrically with the right ROI. For comparison of the data between the different mice, the increase in fluorescence intensity at 120 min after the start of ischaemia on the right ischaemic side was taken as 100%, and the data at the other time points and the data on the non‐ischaemic side were expressed relative to this value. In the control group of mice, a rubber cuff was set around the right thigh, but no pressure was applied.

### Intrathecal application of drugs

For flavoprotein fluorescence imaging, the spinal cord with the intact dura mater was exposed at the L5–L6 level, and 5 μl of 100 mM l‐NAME (FUJIFILM Wako, Osaka, Japan), 2 mM NOR3 ((±)‐(*E*)‐4‐ethyl‐2‐[(*E*)‐hydroxyimino]‐5‐nitro‐3‐hexanamide, FUJIFILM Wako) or saline was intrathecally injected using a hypodermic needle of 30 gauge under direct visual control. When the effects of group II mGluRs on ischaemia‐induced NO production were examined, 5 μl of 10 nM LY354740 (Santa Cruz Biotechnology, Santa Cruz, CA, USA), an agonist of group II mGluRs, was intrathecally injected 30 min before visualization of NO production. For behavioural experiments, 5 μl of 2 mM NOR3 or saline was blindly injected into the intrathecal space at the L5–L6 level of the non‐anesthetized mice. In the latter case, the position of the injection was verified by tail flick responses elicited by insertion of the needle (Hylden & Wilcox, 1980). In preliminary behavioural experiments, 5.0 μl of 1 mM NOR3 showed no significant effects.

### Estimation of mechanical hypersensitivity

The mechanical thresholds for hindpaw‐withdrawal reflex were measured using the von Frey filaments (Amaya *et al*. 2009). The forces produced by the von Frey filaments were between 0.04 and 2 g. Mice were placed separately in a transparent plastic box with a mesh floor, and accustomed to the state for 30 min. The thresholds were determined based on the minimal force that induced a hindpaw‐withdrawal reflex at least twice in 10 trials. The thresholds were measured before and 30–180 min after intrathecal application of 5 μl of 2 mM NOR3 or saline.

### Immunohistochemistry

Mice were deeply anaesthetized with isoflurane, and perfused with a buffered 10% formaldehyde solution (Mildform 10N; FUJIFILM Wako). The spinal cords obtained from the lumbar enlargements were collected as soon as possible. The tissues were immersed in the same fixative at 4°C for 4 h or overnight, and equilibrated in 20% sucrose overnight at 4°C before cryoprotection. Sections with a thickness of 10 μm were prepared using a cryostat microtome (CM1520, Leica Microsystems), mounted on APS‐coated slide glasses (S8441; Matsunami, Osaka, Japan), and stored at –70°C until use. Sections were washed three times with 0.1 M PBS (pH 7.6) for 10 min, then treated with 2% goat serum for 60 min. The sections were primarily incubated with a goat anti‐nNOS antibody (1:100, ab1376; Abcam, Cambridge, UK) and a rabbit anti‐mGluR2 and anti‐mGluR3 antibody (1:100, ab6438; Abcam) for 2 days at 4°C. After washing, sections were secondarily incubated with a donkey anti‐goat IgG H&L conjugated with Alexa Fluor488 (to visualize nNOS; 1:1000, ab150129; Abcam), and a donkey anti‐rabbit IgG conjugated with rhodamine (to visualize mGluR2 and mGluR3; 1:2000 AP182R; EMD Millipore, Burtlington, MA, USA) overnight at 4°C. After washing, the sections were mounted using a medium containing DAPI (H‐1200; Vector Laboratories, Burlingame, CA, USA).

### *In situ* hybridization

Double staining with *in situ* hybridization and immunohistochemistry was performed on frozen sections, as previously described (Toda *et al*. 2018). To generate the probes for *in situ* hybridization, rat *mGluR2* and *mGluR3* plasmids were obtained from RIKEN BRC (Tsukuba, Japan). Digoxigenin‐labelled probes were generated using the appropriate polymerases (T7 or T3 polymerases). Sense probes were used as negative controls. Sections were washed twice with 0.01 M PBS for 5 min, treated with 1 μg/ml proteinase K in 50 mM Tris‐HCl at pH 7.6 and 5 mM EDTA for 60 min, and then rinsed in 0.01 M PBS. After fixation in 4% paraformaldehyde in 0.01 M PBS, and acetylation in 0.1 M triethanolamine at pH 8.0 containing 0.25% acetic anhydride for 10 min, the sections were prehybridized at 65°C for 3 h with a hybridization solution that contained 50% formamide, 0.75 M NaCl, 0.075 M sodium citrate in diethylpyrocarbonate‐treated water, 0.2 mg/ml yeast tRNA, 0.1 mg/ml heparin, 1× Denhardt's solution, 0.2% Tween 20 and 5 mM EDTA. Then, the sections were incubated with a hybridization solution containing a diluted digoxigenin‐labelled RNA probe at 65°C overnight. Hybridized sections were washed twice with 50% formamide, 0.15 M NaCl, 0.015 M sodium citrate in diethylpyrocarbonate‐treated water at 65°C for 15 min (first time) and 30 min (second time), and finally with 0.15 M NaCl, 0.015 M sodium citrate in diethylpyrocarbonate‐treated water at 65°C for 30 min. The sections were washed twice in maleic acid buffer (0.1 M maleic acid at pH 7.5, 0.15 M NaCl and 0.1% Tween 20) for 30 min at room temperature and incubated with alkaline phosphatase‐conjugated sheep anti‐digoxigenin antibody diluted with 0.5% skimmed milk (1: 2000; Roche Diagnostics, Manheim, Germany) overnight at 4°C. Then, they were washed three times with PBS for 30 min each, and incubated with the colour development solution [50 μg/ml 4‐nitro blue tetrazolium chloride (NBT) and 175 μg/ml 5‐bromo‐4‐chloro‐3‐indolyl‐phosphate (BCIP); Roche Diagnostics] in alkaline phosphatase buffer (0.1 M Tris‐HCl at pH 9.5, 0.05 M MgCl_2_, 0.1 M NaCl and 0.1% Tween 20) for 3–10 h in the dark. After *in situ* hybridization, immunohistochemistry was performed using a goat anti‐nNOS antibody (1:100, ab1376; Abcam) and HRP‐conjugated anti‐goat antibody as a secondary antibody (1: 200; MBL, Nagoya, Japan).

### Statistical analysis

Unpaired data obtained from experimental mice administered with l‐NAME or NOR3 and control mice administered with saline vehicle were evaluated using a two‐way ANOVA in Easy R, a free software program for statistical analysis (Kanda, 2013). The data obtained from nNOS knockout mice were compared with those obtained from wild‐type mice of the same age and sex. In *post hoc* analyses, unpaired data obtained in different groups of mice were evaluated using the *t* test with the Bonferroni correction for multiple comparisons. Paired data obtained on the ischaemic side and the non‐ischaemic side of the spinal cord of the same mice were evaluated using a paired *t* test. Unpaired data obtained from different mice were evaluated using a *t* test. Values presented in the figures represent the mean and SEM in the groups of mice, unless otherwise specified. The number of mice in each group was adjusted to be between 5 and 10 to obtain significant results within this range. P values less than 0.05 are usually not shown. The difference in the frequency of *mGluR2* or *mGluR3* expression in nNOS‐positive neurons was evaluated by using the χ^2^‐test in Excel.

## Results

### NO‐mediated cortical spreading potentiation induced by hindpaw ischaemia

**Figure 1:**
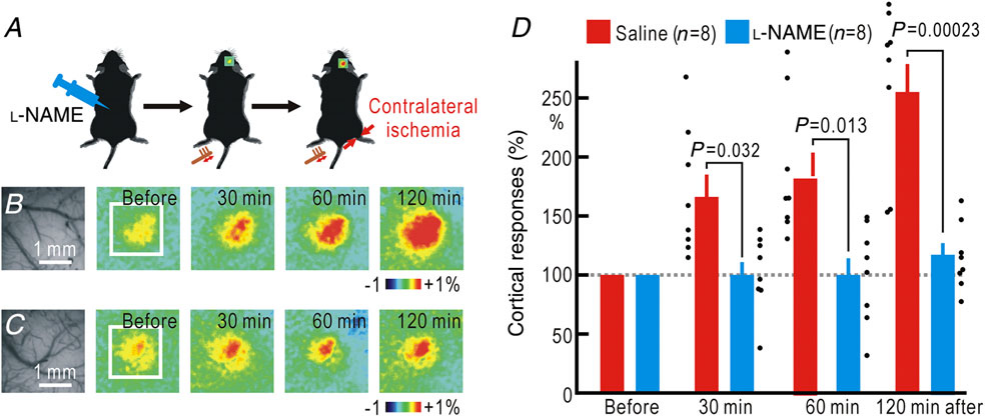
Cortical spreading potentiation during right hindpaw ischaemia with or without the spinal application of l‐NAME *A*, experimental method. *B*, cortical responses in the right somatosensory cortex elicited by brush vibration applied to the sole of the left hindpaw. In these mice, 5 μl saline was intrathecally applied to the spinal cord before the recordings. Original and pseudocolour images in Δ*F*/*F*_0_ of the right somatosensory cortices are shown. The time after the onset of ischaemia in the right hindpaw is shown in each pseudocolour image. The white square in the left‐most pseudocolour image represents the ROI for the measurement of the response amplitude in Δ*F*/*F*_0_. The pseudocolour scale shows the percentages of Δ*F*/*F*_0_. *C*, cortical somatosensory responses elicited by hindpaw stimulation before and after the onset of ischaemia in the right hindpaw. In these mice, l‐NAME (5 μl, 100 mM) was applied to the spinal cord. Original and pseudocolour images in Δ*F*/*F*_0_ are shown. *D*, relative amplitudes of the cortical responses normalized by those recorded before the onset of ischaemia. Mean and SEM are shown. Individual data are also shown by dots. The scale in the bar graphs represents the relative amplitude of the responses as a percentage. [Color figure can be viewed at http://wileyonlinelibrary.com]

Cortical responses to hindpaw stimulation are bilaterally potentiated by hemilateral hindpaw ischaemia (Watanabe *et al*. 2015). To test the hypothesis that this cortical spreading potentiation is mediated by endogenous NO, we administered 5 μl of 100 mM l‐NAME, an inhibitor of NOS, to the spinal cord (Fig. 1 *A*). When saline was administered to the spinal cord as a control, cortical spreading potentiation appeared at 30 min after the onset of hindpaw ischaemia, and the potentiation persisted for 120 min during the ischaemia (Fig. 1 *B*–*D*). The amplitude of cortical potentiation induced by 120 min of ischaemia was 254 ± 27% (*n* = 8) when saline was administered, and 134 ± 6% (*n* = 8) or 114 ± 10% (*n* = 8) when 50 or 100 mM l‐NAME was administered, respectively. Therefore, we used 100 mM l‐NAME in the present study. A two‐way ANOVA showed significant effects of 100 mM l‐NAME (*P* = 1.9 × 10^−8^) and time after the onset of ischaemia (*P* = 0.0099), but not in the interaction. In the *post hoc* analysis, significant differences in the potentiation were observed between the two groups at 30, 60 and 120 min after the onset of ischaemia (Fig. 1 *D*).

**Figure 2:**
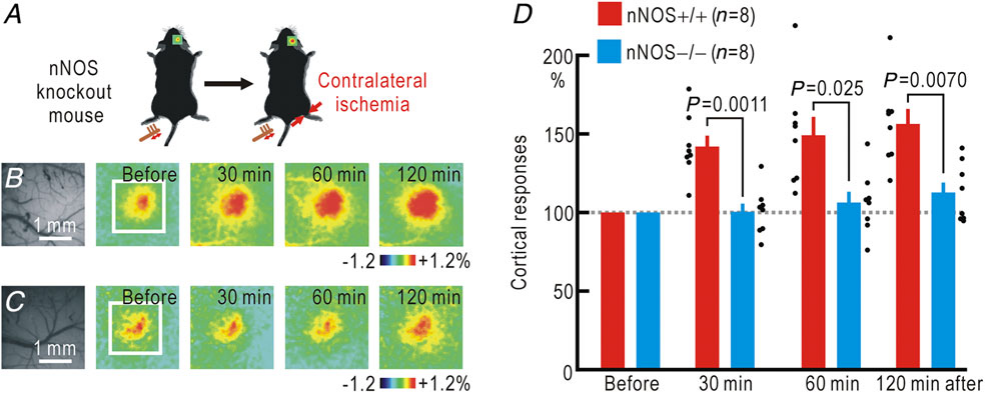
Cortical potentiation during hindpaw ischaemia contralateral to the stimulated hindpaw in wild‐type and nNOS knockout mice *A*, experimental method. *B*, cortical responses elicited by hindpaw stimulation in wild‐type (nNOS+/+) mice before and after the onset of hindpaw ischaemia. Original and pseudocolour images in Δ*F*/*F*_0_ of the right somatosensory cortices are shown. *C*, cortical somatosensory responses elicited by hindpaw stimulation in knockout (nNOS−/−) mice before and after the onset of ischaemia in the right hindpaw. Original and pseudocolour images in Δ*F*/*F*_0_ are shown. *D*, relative amplitudes of the cortical responses normalized by those recorded before the onset of hindpaw ischaemia. [Color figure can be viewed at http://wileyonlinelibrary.com]

There are three types of NOS (Bredt & Snyder, 1992). Because cortical potentiation was induced within 30 min of hindpaw ischaemia, the involvement of inducible NOS (iNOS) is unlikely. Because nNOS is present in the dorsal horn of the spinal cord (Zhang *et al*. 1993; Reuss & Reuss, 2001), nNOS is likely to be responsible for inducing the cortical potentiation. To confirm this hypothesis, experiments were carried out in mice lacking nNOS (Fig. 2 *A*). In wild‐type mice, hindpaw ischaemia in the non‐stimulated side induced cortical potentiation (Fig. 2 *B*, *D*). However, almost no potentiation was observed following ischaemia in the nNOS knockout mice (Fig. 2 *C*, *D*). A two‐way ANOVA showed significant effects of nNOS knockout (*P* = 1.4 × 10^−7^), but not of the time after the onset of ischaemia and their interaction. In the *post hoc* analysis, significant differences in potentiation were observed between the two groups at 30, 60 and 120 min after the onset of ischaemia (Fig. 2 *D*).

**Figure 3:**
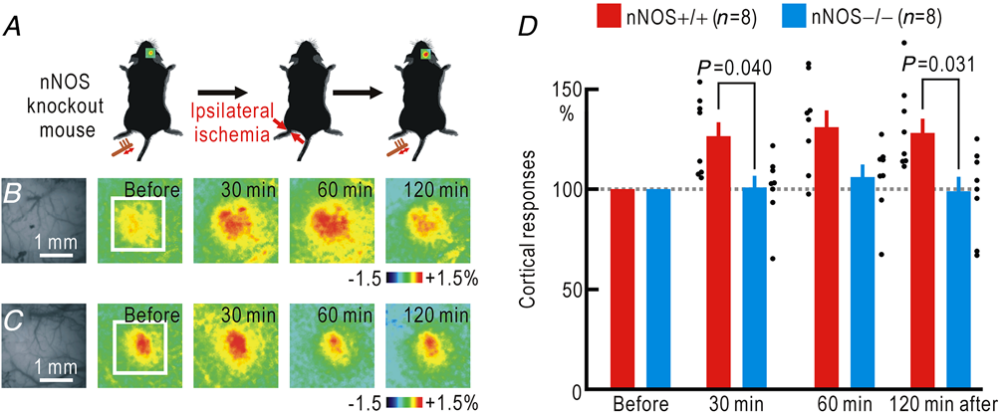
Cortical potentiation after ischaemia in the stimulated hindpaw in wild‐type and nNOS knockout mice *A*, experimental method. *B*, right cortical responses elicited by left hindpaw stimulation in wild‐type (nNOS+/+) mice before and after ischaemia in the left hindpaw for 30 min. Original and pseudocolour images in Δ*F*/*F*_0_ of the right somatosensory cortices are shown. The time after termination of hindpaw ischaemia is shown in each pseudocolour image. *C*, right cortical responses elicited by left hindpaw stimulation in knockout (nNOS−/−) mice before and after ischaemia in the left hindpaw for 30 min. *D*, relative amplitudes of the cortical responses normalized by those recorded before hindpaw ischaemia. [Color figure can be viewed at http://wileyonlinelibrary.com]

Somatosensory responses to stimuli applied to an ischaemic hindpaw cannot be elicited because of ischaemic conduction block of the peripheral nerves. However, comparison of the somatosensory responses before ischaemia and after recovery from ischaemia reveals that cortical potentiation also occurs on the ischaemic side (Watanabe *et al*. 2015). It is very likely that the two types of potentiation in both sides share common mechanisms. To confirm this hypothesis, we used nNOS knockout mice and compared the somatosensory responses before ischaemia and after recovery from ischaemia to test whether cortical potentiation in the responses to stimuli applied to the hindpaw was induced by ischaemia (Fig. 3 *A*). In wild‐type mice, potentiation was observed 30 min after hindpaw ischaemia was terminated, and this potentiation persisted for 120 min (Fig. 3 *B*, *D*). However, no such potentiation was recorded in the nNOS knockout mice (Fig. 3 *C*, *D*). A two‐way ANOVA showed significant effects of nNOS knockout (*P* = 1.9 × 10^−5^), but not of the time after the onset of ischaemia or their interaction. In the *post hoc* analysis, significant differences in potentiation were observed between the two groups at 30 and 120 min after the onset of ischaemia (Fig. 3 *D*). These results suggest strongly that NO induces cortical spreading potentiation on both sides, possibly due to diffusion of NO within the spinal cord.

### NO‐mediated spinal spreading potentiation induced by hindpaw ischaemia

**Figure 4:**
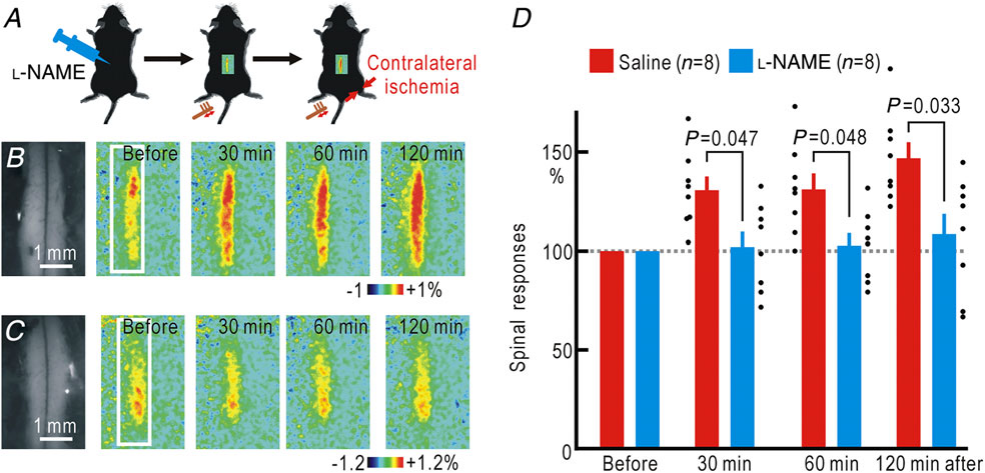
Spinal spreading potentiation during right hindpaw ischaemia with or without spinal application of l‐NAME *A*, experimental method. *B*, spinal responses in the left spinal cord elicited by brush vibration applied to the sole of the left hindpaw. In these mice, 5 μl saline was applied to the spinal cord before the recordings. Original and pseudocolour images in Δ*F*/*F*_0_ of the spinal cord are shown. The time after the onset of ischaemia in the right hindpaw is shown in each pseudocolour image. The white rectangle in the left‐most pseudocolour image represents the ROI for the measurement of the response amplitude in Δ*F*/*F*_0_. *C*, spinal responses elicited by left hindpaw stimulation before and after the onset of ischaemia in the right hindpaw. In these mice, l‐NAME (5 μl, 100 mM) was applied to the spinal cord. Original and pseudocolour images in Δ*F*/*F*_0_ are shown. *D*, relative amplitudes of the spinal responses normalized by those recorded before the onset of hindpaw ischaemia. [Color figure can be viewed at http://wileyonlinelibrary.com]

Spatial spread of the somatosensory potentiation induced by hindpaw ischaemia is observed not only at the cortical but also at the spinal level (Watanabe *et al*. 2015). Therefore, it is very likely that the spinal spreading potentiation is also mediated by NO. To confirm this hypothesis, we investigated the spinal responses using flavoprotein fluorescence imaging. After spinal application of saline or l‐NAME into the intrathecal space, the surface of the spinal cord was uncovered, and the spinal cord responses to left hindpaw stimulation were recorded before and during ischaemia of the opposite right hindpaw (Fig. 4 *A*). When saline was administered, spinal potentiation appeared 30 min after the onset of the hindpaw ischaemia, and the potentiation persisted for at least 2 h (Fig. 4 *B*, *D*). However, no such potentiation was observed in the mice treated with l‐NAME (Fig. 4 *C*, *D*). A two‐way ANOVA showed significant effects of l‐NAME (*P* = 1.7 × 10^−5^), but not of the time after the onset of ischaemia or their interaction. In the *post hoc* analysis, significant differences in potentiation were observed between the two groups at 30, 60 and 120 min after the onset of ischaemia (Fig. 4 *D*).

**Figure 5:**
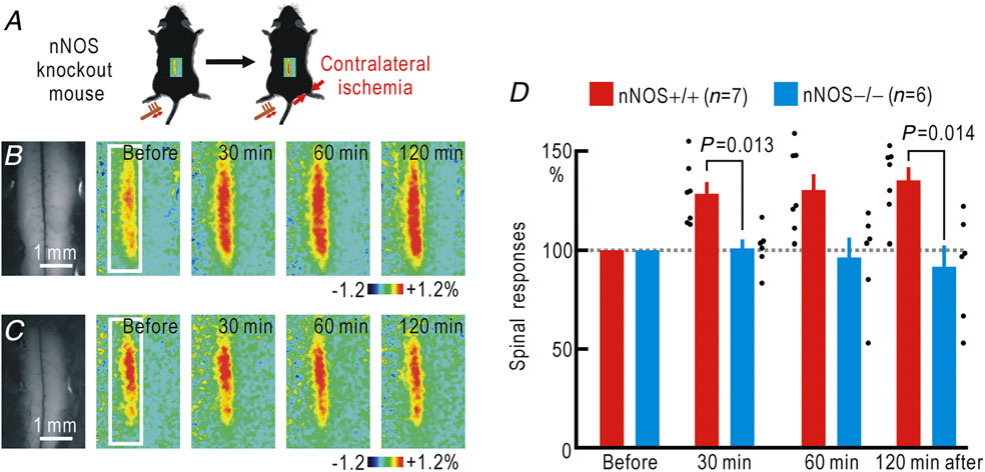
Spinal spreading potentiation during right hindpaw ischaemia in wild‐type and nNOS knockout mice *A*, experimental method. *B*, spinal responses elicited by left hindpaw stimulation in wild‐type (nNOS+/+) mice before and after the onset of ischaemia in the right hindpaw. Original and pseudocolour images in Δ*F*/*F*_0_ of the spinal cord are shown. *C*, spinal responses elicited by left hindpaw stimulation in knockout (nNOS−/−) mice before and after the onset of ischaemia in the right hindpaw. Original and pseudocolour images in Δ*F*/*F*_0_ are shown. *D*, relative amplitudes of the spinal responses normalized by those recorded before the onset of ischaemia. [Color figure can be viewed at http://wileyonlinelibrary.com]

Cortical spreading potentiation during hindpaw ischaemia was not observed in nNOS knockout mice. Therefore, we tested whether spinal spreading potentiation was blocked in nNOS knockout mice (Fig. 5 *A*). The amplitudes (Δ*F*/*F*_0_) of the spinal responses to hindpaw stimulation were 0.56 ± 0.4% (*n* = 8) in wild‐type mice and 0.64 ± 0.5% (*n* = 7) in nNOS knockout mice. The difference was not statistically significant (*P* = 0.13). Even though spinal spreading potentiation was observed in control wild‐type mice (Fig. 5 *B*, *D*), no such potentiation was observed in nNOS knockout mice (Fig. 5 *B*, *D*). A two‐way ANOVA showed significant effects of nNOS knockout (*P* = 4.8 × 10^−6^), but not of the time after the onset of ischaemia or their interaction. In the *post hoc* analysis, significant differences in potentiation were observed between the two groups at 30 and 120 min after the onset of ischaemia (Fig. 5 *D*). The differences between both groups of mice were significant between 30 min and 2 h after the onset of ischaemia (Fig. 5 *D*). Taken together, these results suggest strongly that NO‐mediated cortical spreading potentiation induced by hindpaw ischaemia is a result of NO‐mediated spreading potentiation at the spinal cord level.

### Cortical potentiation and mechanical hypersensitivity induced by exogenous NO

**Figure 6:**
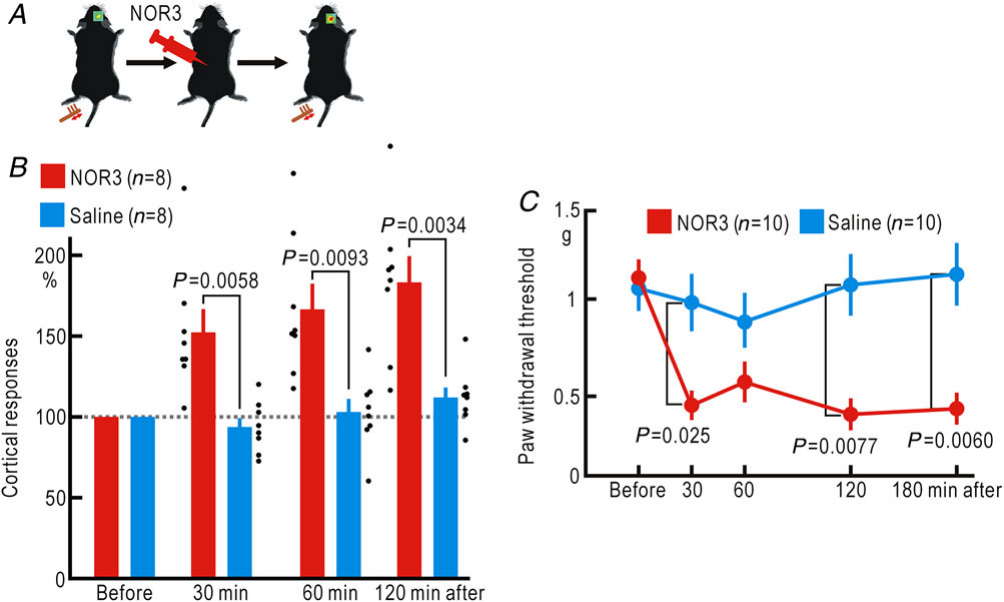
Cortical potentiation and mechanical hypersensitivity induced by spinal application of NOR3 *A*, experimental method. *B*, relative amplitudes of the cortical responses normalized by those recorded before spinal application of 5 μl saline or 2 mM NOR3. The abscissa shows the time after the spinal application of saline or NOR3. *C*, mechanical threshold of the hindpaw withdrawal reflex measured by the von Frey test before spinal application of 5 μl saline or 2 mM NOR3. [Color figure can be viewed at http://wileyonlinelibrary.com]

The left and right sides of the spinal cord are connected via neural circuits (Fitzgerald, 1982; Koltzenburg *et al*. 1999). If the spatial spread of cortical/spinal potentiation is attributed to neural activities mediated via such neural circuits, the presence of NO within the spinal cord may not be enough to induce somatosensory potentiation. To test this possibility, we investigated whether cortical responses are potentiated after application of NOR3, an NO donor, into the spinal intrathecal space (Fig. 6 *A*). As a result, cortical potentiation was observed between 30 min and 2 h after application of NOR3 (Fig. 6 *B*). Such changes were not observed after intrathecal administration of saline. A two‐way ANOVA showed significant effects of NOR3 (*P* = 5.6 × 10^−8^), but not of the time after application of NOR3 or their interaction. In the *post hoc* analysis, significant differences in potentiation were observed between the two groups at 30, 60 and 120 min after application of NOR3 (Fig. 6 *B*).

Cortical potentiation after hindpaw ischaemia is accompanied by mechanical hypersensitivity (Watanabe *et al*. 2015). Therefore, mechanical hypersensitivity may also occur after spinal application of NOR3. When we investigated the hindpaw withdrawal reflex with the von Frey test, the mechanical threshold decreased 30 min after the spinal application of NOR3 and remained low until 3 h after the application (Fig. 6 *C*). On the other hand, the mechanical threshold was unchanged when saline was administered to the spinal cord. A two‐way ANOVA showed significant effects of NOR3 (*P* = 1.9 × 10^−7^), the time after application of NOR3 (*P* = 0.015), and their interaction (*P* = 0.015). In the *post hoc* analysis, significant differences in potentiation were observed between the two groups at 30, 120 and 180 min after application of NOR3 (Fig. 6 *C*). These results indicate that the presence of NO is enough to induce somatosensory spreading potentiation and mechanical hypersensitivity.

### Visualization of spinal NO distribution during unilateral hindpaw ischaemia

**Figure 7:**
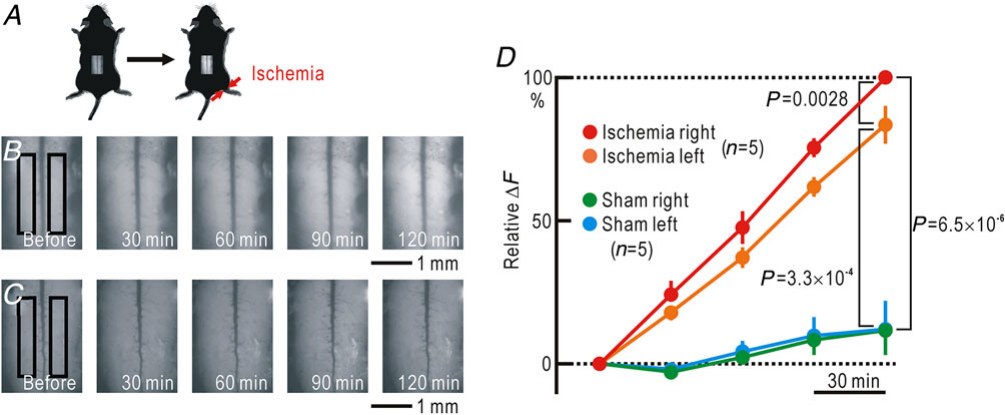
Visualized spinal NO production during right hindpaw ischaemia using DAF‐FM *A*, experimental method. *B*, images of the spinal cord covered with 2% agarose gel containing 20 μM DAF‐FM. The time after the onset of ischaemia in the right hindpaw is shown in each image. The intensity of the fluorescence is shown on an arbitrary grey scale. The black rectangles represent ROIs for measurement of fluorescence intensity. *C*, images of the spinal cord covered with 2% agarose gel containing DAF‐FM. In this experiment, sham treatment without pressure was applied to the right hindpaw. *D*, relative amplitudes of the increases in fluorescence normalized by those recorded 2 h after the onset of right hindpaw ischaemia in the right side of the spinal cord. [Color figure can be viewed at http://wileyonlinelibrary.com]

Spinal application of l‐NAME prevented cortical potentiation and spinal potentiation during hindpaw ischaemia. These results strongly suggest that NO is produced in the spinal cord during hindpaw ischaemia possibly in the ischaemic side, and diffuses to the non‐ischaemic side. To confirm this hypothesis, we visualized the endogenous NO produced in the mouse spinal cord. We used DAF‐FM, which binds to NO and is converted to a fluorescent form (Kojima *et al*. 1999; Mabuchi *et al*. 2003). DAF‐FM was mixed into an agarose gel covering the spinal cord surface, and the changes in fluorescence intensity during hindpaw ischaemia were recorded (Fig. 7 *A*). In mice with hindpaw ischaemia, gradual increases in fluorescence intensity during hindpaw ischaemia were observed on the ischaemic side (Fig. 7 *B*). The fluorescence intensity increased almost linearly with time (Fig. 7 *D*). Increases in fluorescence intensity were observed not only on the ischaemic side but also on the non‐ischaemic side. However, when compared at 2 h after the onset of ischaemia, the fluorescence increase on the ischaemic side was significantly stronger than that on the non‐ischaemic side (Fig. 7 *D*). In sham operated mice, only small increases in fluorescence intensity were observed in the spinal cord. These results suggest strongly that NO is continuously produced in the spinal cord on the ischaemic side during hindpaw ischaemia, and a considerable amount of the NO produced rapidly diffuses to the non‐ischaemic side.

### Interaction between mGluR2 and nNOS in spinal neurons

Activation of group II mGluRs, mGluR2 and mGluR3 (Tanabe *et al*. 1992), is known to alleviate neuropathic pain (Simmons *et al*. 2002; Jones *et al*. 2005; Osikowicz *et al*. 2013). In our previous studies, spinal application of LY354740 (10 nM, 5 μl), an agonist of group II mGluRs, blocked cortical potentiation induced by partial cutting of the peripheral nerves (Komagata *et al*. 2011) and cortical/spinal potentiation induced by hindpaw ischaemia (Watanabe *et al*. 2015). Therefore, we tested the effect of LY354740 (10 nM, 5 μl) on the changes in spinal DAF‐FM fluorescence induced by hindpaw ischaemia. DAF‐FM fluorescence was increased by 53 ± 7% (*n* = 5) during a 120 min period of ischaemia, while it was slightly decreased by 15 ± 5% (*n* = 6) in the presence of LY354740. As these values were significantly different (*P* = 5.0 × 10^−5^), these findings suggest strongly that the role of group II mGluRs on cortical/spinal potentiation is mediated by NO production.

**Figure 8:**
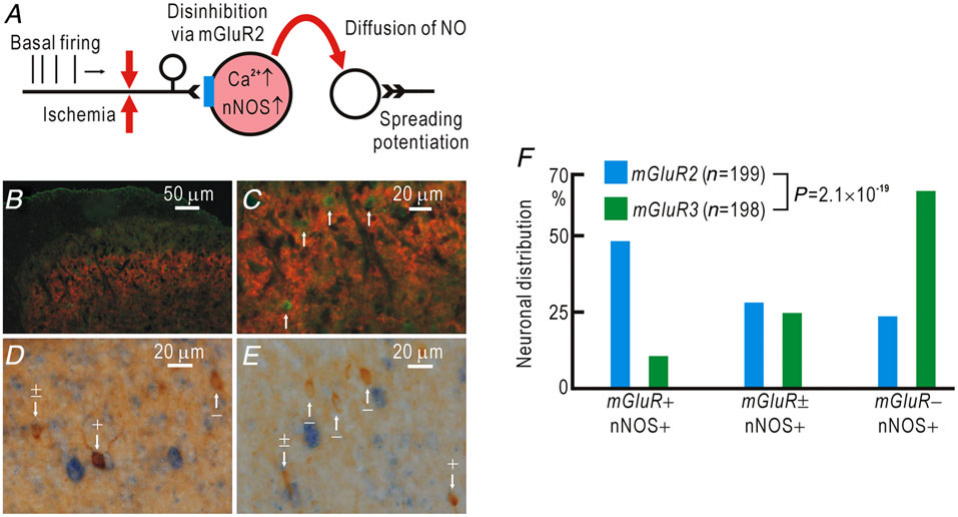
Colocalization of mGluR2 and nNOS in spinal neurons *A*, hypothetical relationship between signalling pathways mediated via mGluR2 and NO. *B*, immunohistochemical staining of group II mGluRs (mGluR2 and mGluR3, red) and nNOS (green). Colocalization of group II mGluRs and nNOS was observed in the superficial laminae of the dorsal horn. *C*, the same image shown in *B* at higher magnification. The cell bodies with nNOS (white arrows) were not stained with antibodies for group II mGluRs. *D*, immunostaining for nNOS (brown) and *in situ* hybridization of mRNA for *mGluR2* (blue). Some cell bodies with nNOS were clearly (+), faintly (±) or not stained (–) with mRNA for *mGluR2*. White arrows show examples. *E*, immunostaining for nNOS (brown) and *in situ* hybridization of mRNA for *mGluR3* (blue). *F*, relative distribution of nNOS‐positive spinal neurons that were clearly (+), faintly (±) or not stained (–) with mRNA for *mGluR2* or *mGluR3*. [Color figure can be viewed at http://wileyonlinelibrary.com]

A simple explanation of the interaction between mGluR2 and NO production is that group II mGluRs and nNOS co‐localize in spinal neurons that receive glutamatergic inputs from peripheral nerves, so that ischaemic conduction block of basal firing in Aβ afferents (Komagata *et al*. 2011) produces disinhibition via group II mGluRs, resulting in an elevation of nNOS activity (Fig. 8 *A*). Although other possible interactions between group II mGluRs and nNOS cannot be excluded, the distribution of group II mGluRs and nNOS in the spinal cord is an important clue for understanding this relationship. We performed immunostaining of group II mGluRs and nNOS (Fig. 8 *B*, *C*). Group II mGluRs were enriched in the superficial laminae of the dorsal horn (Fig. 8 *B*). Cellular bodies with immunoreactivity to nNOS were also distributed in the superficial laminae of the dorsal horn (Fig. 8 *B*, *C*). Unlike nNOS, group II mGluRs are distributed mainly outside of the cell bodies (Fig. 8 *C*), possibly in the dendritic postsynaptic sites or presynaptic nerve terminals.

Our immunostaining experiments did not reveal whether group II mGluRs co‐localize with nNOS in the same spinal neurons or not. Furthermore, the antibody used in our experiments binds to both mGluR2 and mGluR3 in group II mGluRs, and it is not clear which of them coexists in nNOS‐positive spinal neurons. Therefore, we separately visualized the mRNAs of *mGluR2* and *mGluR3* by *in situ* hybridization (Fig. 8 *D*, *E*). Approximately half of the nNOS‐positive neurons were clearly stained with the *mGluR2* mRNA while only approximately 10% of nNOS‐positive neurons were clearly stained with the *mGluR3* mRNA (Fig. 8 *F*). These results are compatible with the possibility shown in Fig. 8 *A* and suggest that spinal dorsal horn neurons expressing both mGluR2 and nNOS have a crucial role in the cortical/spinal spreading potentiation induced by hindpaw ischaemia.

## Discussion

### Technical merits of the present mouse model

In the present study, we used a mouse model to test the hypothesis that the spatial spread of cortical/spinal potentiation induced by nerve conduction block is attributed to NO, a diffusible messenger (Garthwaite & Boulton, 1995; Garthwaite, 2016). One of the merits of our mouse model is that transcranial imaging of cortical responses using flavoprotein fluorescence is possible. Activity‐dependent fluorescence changes derived from mitochondrial flavoproteins are sufficiently rapid to capture the dynamic neural activity *in vivo* (Shibuki *et al*. 2003; Reinert *et al*. 2004). We measured the amplitudes of the flavoprotein fluorescence responses at 0.6–1.0 s after the stimulus onset. The haemodynamic responses are induced after this time window (Kitaura *et al*. 2007). A close relationship between the neuronal responses and fluorescence imaging has been demonstrated in previous studies (Sibuki *et al* 2003; Reinert *et al*. 2004; Tohmi *et al*. 2006). Various types of cortical plasticity including somatosensory potentiation induced by hindpaw ischaemia have been demonstrated using this method (Takahashi *et al*. 2006; Tohmi *et al*. 2006; Komagata *et al*. 2011; Yoshitake *et al*. 2013; Watanabe *et al*. 2015). We also recorded the potentiation of spinal responses during hindpaw ischaemia, as previously reported (Watanabe *et al*. 2015).

We succeeded in visualizing endogenous production of NO in the spinal cord *in vivo* using DAF‐FM, which emits green fluorescence when combined with NO in spinal slices (Mabuchi *et al*. 2003; Xu *et al*. 2006). We were also able to record the signals derived from DAF‐FM in the spinal cord of anaesthetized mice for the first time. The linear increase in the intensity of the fluorescence derived from DAF‐FM indicated that NO was constantly produced during hindpaw ischaemia. A fluorescence increase was also recorded on the opposite side approximately 1 mm from the ischaemic side. The intensity on the non‐ischaemic side was significantly weaker than that on the ischaemic side, suggesting that NO is produced on the ischaemic side and quickly diffuses into the non‐ischaemic side. Diffusion of NO has also been observed in spinal slices: when a spinal slice loaded with DAF‐FM is stimulated with NMDA, fluorescence increases derived from NO are similarly observed in both superficial and deep laminae, while only superficial laminae are enriched with NOS (Xu *et al*. 2006).

### Involvement of NO in somatosensory potentiation

NO is a diffusible mediator that is characterized by a small molecular weight and no electrical charge. In addition to its vasodilatory effect, NO has important roles in the induction of various types of synaptic plasticity in the CNS (Garthwaite & Boulton, 1995; Garthwaite, 2016). It has also been shown that NO is involved in the pathogenesis of neuropathic pain at the spinal cord level (Wu *et al*. 2001; Schmidtko *et al*. 2009; Tanabe *et al*. 2009). NO promotes the generation of inflammatory mediators (Salvemini *et al*. 1993) and is known to induce neuropathic pain via various messengers (Schmidtko *et al*. 2009). Several studies have shown that NO produced by nNOS is important for neuropathic pain (Cope *et al*. 2010; Roh *et al*. 2011). On the other hand, other studies have reported that iNOS expression is important for neuropathic pain (De Alba *et al*. 2006; Quan *et al*. 2013). In the present study, spinal application of NOR3 produced cortical potentiation and mechanical hypersensitivity, thus indicating that the presence of spinal NO is enough to produce cortical potentiation and mechanical hypersensitivity, regardless of the source of NO. As we focused on the acute changes occurring within 30 min after the onset of ischaemia, the importance of nNOS was clearly demonstrated. However, our results do not exclude the possibility that the expression of iNOS, which is increased during inflammation in the chronic phase of neuropathic pain, plays an important role in the pathogenesis of neuropathic pain. Peripheral nerve injuries result in neuropathic pain and neuroplasticity not only on the side of the injury but also on the contralateral side in the chronic phase (Milligan *et al*. 2003). This expansion in the pain areas is attributable to invasion of the inflammatory process and the diffusion of inflammatory substances (Milligan *et al*. 2003; Racz *et al*. 2008; Carlton *et al*. 2009). The present results raise the possibility that NO‐mediated potentiation may contribute to the spread of neuropathic pain beyond the injured sites in the chronic phase of neuropathic pain, as well as in the acute phase. Furthermore, the spread, and consequently the non‐specific NO‐mediated spinal potentiation induced by ischaemia may modify heat or cold pain sensitivity, as it induces mechanical hypersensitivity.

### Mechanisms underlying NO‐mediated spreading potentiation

When peripheral nerves lose excitability by injuries or ischaemia, spontaneous firing of the peripheral nerve fails to reach spinal neurons (Komagata *et al*. 2011; Watanabe *et al*. 2015). If NO is produced in the spinal neurons that fail to receive spontaneous inputs from peripheral nerves, it can diffuse to the surrounding neurons to produce potentiation in a non‐specific manner. Such a mechanism may explain how various spinal sites exhibit potentiation after conduction block of peripheral nerves. Somatosensory potentiation is induced within 2–3 h after partial nerve cutting (Komagata *et al*. 2011) and within 30 min after recovery from hindpaw ischaemia (Watanabe *et al*. 2015). Hindpaw ischaemia may produce a conduction block in more nerve fibres than partial nerve cutting, so that NO is produced in more spinal neurons to induce a rapid potentiation. In the present study, we observed that both nNOS and group II mGluR are present in the superficial laminae of the dorsal horn. Furthermore, the mRNA of *mGluR2* was present in approximately half of the nNOS‐positive cell bodies of spinal neurons in this area. Activation of group II mGluR inhibits neurotransmitter release in the presynaptic terminals (Goudet *et al*. 2009) and hyperpolarizes the postsynaptic neurons by opening potassium channels (Irie et al. 2006; Lee & Sherman, 2010), thus suppressing Ca^2+^ influx (Koga *et al*. 2010). Therefore, disinhibition of group II mGluR effects by conduction block of peripheral nerves can lead to a sustained increase in intracellular Ca^2+^ levels, which maintains nNOS activity at a plateau level. It has also been reported that a rise in intracellular Ca^2+^ levels of spinal lamina I neurons is enough to induce synaptic long‐term potentiation in the absence of any presynaptic stimulation (Naka *et al*. 2013).

The gate control theory assumes that nociceptive spinothalamic tract neurons receive innocuous afferent inputs from various sources, and the responses to innocuous afferents are normally inhibited via interneurons in the superficial laminae of the dorsal horn (Melzack & Wall, 1965; Daniele & MacDermott, 2009). However, nociceptive spinothalamic tract neurons show increased responsiveness or potentiation to innocuous mechanical stimuli in animals with injured nerves (Paleček *et al*. 1992). These changes after nerve injury are explained by a reduced Cl^–^ gradient in these neurons (Sivilotti & Woolf, 1994; Moore *et al*. 2002; Coull *et al*. 2005; Price *et al*. 2009). The decrease in the Cl^–^ gradient is attributable to the downregulation of the neuron‐specific KCl cotransporter (KCC2) in nociceptive spinothalamic tract neurons (Coull *et al*. 2005). KCC2 activity is known to be suppressed by NO (Yassin *et al*. 2014). Thus, spinal neurons that fail to receive spontaneous signals from peripheral nerves can be connected to nociceptive spinothalamic tract neurons that exhibit potentiation to innocuous afferents via diffusion of NO. Even though this explanation does not exclude other possibilities for the mechanisms underlying the initial development of neuropathic pain, it is highly likely that NO plays an essential role in neuropathic pain, as reported in previous studies (Wu *et al*. 2001; Schmidtko *et al*. 2009; Tanabe *et al*. 2009).

### Roles of the spinal cord in neuropathic pain

In the present study, spreading potentiation was observed at the cortical level as well as in the spinal cord. In subjects with neuropathic pain, profound neural plasticity has been reported in the somatosensory cortex (Flor *et al*. 1995; Kim *et al*. 2016), as well as in higher areas such as the anterior cingulate cortex (Xu *et al*. 2008; Zhao *et al*. 2018) and medial prefrontal cortex (Metz *et al*. 2009; Kelly *et al*. 2016; Sang *et al*. 2018). The influence of nerve injuries on the spinal cord occurs initially in the CNS. NO generation in the spinal cord and the accompanying spreading potentiation are the earliest events after peripheral nerves are injured, and may be potentially maintained even in the chronic phase via expression of iNOS (De Alba *et al*. 2006; Quan *et al*. 2013). Therefore, it is natural to assume that the changes in the spinal cord will sequentially spread to other parts of the brain via ascending pathways in the pathogenesis of neuropathic pain. Therefore, NO‐mediated spreading potentiation in the spinal cord is an important target for the treatment of neuropathic pain, because it is the initial event and occurs at the entrance of afferents to higher brain areas involved in neuropathic pain.

## Additional information

### Competing interests

The authors declare no competing financial interests.

### Author contributions

Conception and/or design of the study – T.O., K.S. Acquisition of the data – T.O., M.S., M.H. Analysis or interpretation of the data – T.O., T.W., M.S., Y.K., M.H., H.T., R.H., T.K., H.T., H.B., K.S. Writing of the manuscript – T.O., T.K., K.S. All authors approved the final version of the manuscript and agree to be accountable for all aspects of the work.

### Funding

This work was supported by the Grant‐in‐Aid for Scientific Research (no. 22115011, no.16H01892 to K.S., and no. 17H06692 to T.O.).
